# Electrical Bioimpedance: From the Past to the Future

**DOI:** 10.2478/joeb-2021-0001

**Published:** 2021-11-20

**Authors:** Leigh C Ward

**Affiliations:** 1 School of Chemistry and Molecular Biosciences, The University of Queensland, Brisbane, Australia

**Keywords:** Bioimpedance, electrical impedance, BIA

## Abstract

This year, 2021, marks the “coming of age” for JoEB with its indexing in PubMed Central. It is also a century since some of the earliest studies on tissue impedance. This editorial briefly reviews the time-line of research in the field to mark this occasion.

## Editorial

**Fig 1:**
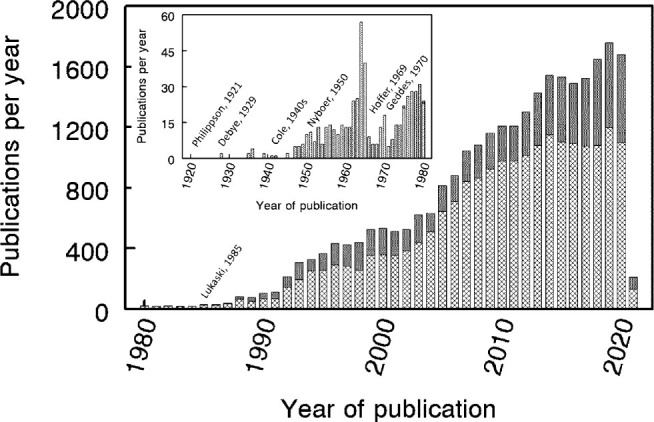
Publications per year in the field of electrical bioimpedance listed on PubMed. “Search query: (“”Bioimpedance”” OR “”bioelectrical impedance”” OR “”electrical impedance”” OR “”electrical bioimpedance”” OR “”Electric impedance”” NOT “”Rheogram”” NOT “”Rheography”” NOT “”Rheographic”” NOT “”Rheograms””) for all publications. The search query was appended with AND (“”Body composition”” OR “”Body water””) to restrict the search to those publications also including reference to body composition. Key 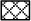 publications excluding body composition; 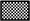 publications including body composition.

It is opportune in 2021 to reflect upon the history of electrical bioimpedance research. While one cannot easily define a starting point for research in the field, 2021 marks one hundred years since the publication by Philippson of “*Les lois de la resistance electrique des tissus vivants*” (*The laws of electrical resistance of living tissue*) [1]. Although interest in the electrical responses of biological (animal tissues) had commenced many years before (e.g., the studies of du Bois-Reymond in the 1850s), the studies of Philippson provide a convenient starting point. Philippson measured the impedance of biological entities, including blood cells and guinea pig muscle and liver of a frequency range of 500 kHz to 3 MHz, presaging modern researches into the frequency response of tissues. Philippson was followed by the pioneering studies of Debye, Fricke, Cole (KS) and others. Unlike the “industrialized” research of today with pressure to publish, the outcomes of these early endeavors were relatively sparse, numbering one or two publications per year, but immensely influential (Fig 1, inset). The late 1940s through to 1980 saw a marked increase in interest, largely stimulated by the work of Nyboer, Geddes and colleagues, with a particular focus on impedance plethysmography for the measurement of cardiovascular function.

The 1980s saw the rapid rise in publications in the field and interest in impedance techniques from various research groups around the world. The initial stimulus for this surge in interest can be largely attributed to the work of Hoffer in 1969, and earlier Thomasset in 1965, who demonstrated that one could estimate total body water from a measurement of whole-body, wrist to ankle, impedance. Until this time, studies of electrical bioimpedance had been largely of theoretical or academic interest rather than of clinical applicability; arguably, impedance plethysmographic measurement of cardiac function had not fully realized its potential and impedance studies were a technology looking for an application. In 1981, Dr William Mills of the U.S. Navy initiated a study to measure the hydration of soldiers at altitude using the impedance technique of Hoffer. For this work, Mills commissioned RJL Systems, a small company (in today's terms a start-up) to design and build a suitable impedance device. RJL Systems had been established a few years earlier by Rudolph Liedtke who had previously worked on Impedance systems with Nyboer at Wayne State University. A little later in 1985, Lukaski and colleagues published what was to become one of the most influential papers in the field. Lukaski demonstrated that the fat-free mass of the human body could be estimated from whole-body impedance measurements using the RJL device. This paper has now been cited over 1400 times and moved what is now known as bioelectrical impedance analysis (BIA) from a laboratory research tool to a clinical tool and ultimately mass consumer technology.

There are now many manufacturers of dedicated impedance devices for body composition assessment. These range from those designed for clinical/medical monitoring and research to simple consumer-orientated devices, the body fatness bathroom scale. In parallel with this technological development has been a marked increase in research activity. Scientific publications related to impedance technology now number more than 1500 per year of which around 40% are associated with body composition measurement (Fig 1 main panel). Many of these publications relate to the use of impedance techniques rather than fundamental research advancing the technology, nevertheless this is extensive. Bioimpedance research is particularly strong in the U.S., UK, Spain, Australia, Sweden, Estonia, Norway, and Brazil. A detailed bibliometric analysis has been published by El Khaled *et al*. [2].

Increasing interest in electrical bioimpedance from the late 1960s gave rise to a series of international conferences now formally organized under the auspices of the International Committee for Promotion of Research in Bio-Impedance (ICPRBI). The first International Conference on Electrical Bioimpedance (ICEBI) was held in late September 1969 and the latest, the 17^th^ in 2019. A brief report of the first meeting was published by Geddes in Science in 1970 [3]; it makes interesting reading. Thirty-three papers were presented in three broad areas clinical use, instrumentation and underlying physiological basis for impedance, areas still familiar to present day researchers. With respect to instrumentation, Geddes highlighted the “*considerable differences of opinion regarding the appropriate type of instrumentation*” and that “*there were advocates for the use of two, three and four terminals (electrodes), crusaders for low and high frequency and adherents of either constant-current or constant-voltage*”. These are issues on which there is now little dissent. Devices, except for some specific applications, are almost exclusively 4-terminal and constant current with frequency ranging devices in common use in body composition analysis. It was decided at the XI ICEBI to establish a bioimpedance society with the International Society for Electrical Bioimpedance formally coming into being when its statutes were approved at the XII ICEBI in 2004.

Bioimpedance research received further impetus in 2010, when the Oslo Bioimpedance Group established the Journal of Electrical Bioimpedance. In the past 12 years, the journal has gone from strength to strength as an international, peer-reviewed open access journal providing a channel for the publication of a wide-range of papers in the broad field of bioimpedance research. It is fitting, in this hundredth year since Philippson's pioneering study, that JoEB has been accepted for indexing in Pubmed Central (https://www.ncbi.nlm.nih.gov/pmc/journals/3936/). This deservedly will bring much greater exposure to the journal and those that publish therein in the future.

Where to now? In 2011, I was invited to present a plenary lecture on the past, present and future of bioimpedance analysis at the International Body Composition meeting, IBC2011. My conclusions were that while bioimpedance technology has a bright future there were unlikely to be major improvements in accuracy (for body composition assessment) but miniaturization of impedance devices and the advent of wearable electrodes and devices would lead to an important role in real-time monitoring of physiological processes. These views still hold a decade later. The increasing application of health monitoring via smart-phone or dedicated device technologies suggest an increasing role for measurement of electrical impedance. The pioneers in the field would be delighted that their interests are still being pursued with vigor and practical applications in human health are being realized.
